# Esophageal Stab Wounds Repaired Endoscopically: A Case Report and Comprehensive Literature Review

**DOI:** 10.7759/cureus.35603

**Published:** 2023-02-28

**Authors:** Ariana R Tagliaferri, Minha Naseer, Shoaib Azam, Gabriel Melki, Matthew A Grossman

**Affiliations:** 1 Internal Medicine, St. Joseph's Regional Medical Center, Paterson, USA; 2 Gastroenterology and Hepatology, St. Joseph's University Medical Center, Paterson, USA; 3 Medicine, St. Joseph's University Medical Center, Paterson, USA; 4 Interventional Gastroenterology, St. Joseph's University Medical Center, Paterson, USA

**Keywords:** endoscopic repair, trauma surgery, trauma, stab wounds, esophagus

## Abstract

Esophageal injuries are typically iatrogenic after endoscopic/surgical procedures, but they are rarely caused by penetrative or blunt trauma. We present a case of patient who suffered multiple stab wounds to the neck and underwent surgical repair for hemorrhagic shock but was ultimately diagnosed and treated successfully via endoscopy for a thoracic esophageal injury. Early detection is imperative and usually diagnosed via contrast studies but less commonly via direct visualization endoscopically. Moreover, endoscopic treatment is also less commonly utilized, even if diagnosed from that modality. Cervical injuries also confer a lower mortality than thoracic injuries.

## Introduction

Esophageal injuries (EIs) are rare diagnoses that can occur in the setting of trauma or with underlying conditions such as achalasia or strictures [1]. The presentation and mechanism of injury differ based on the three regions of the esophagus, in which EIs occur [1]. Injuries to the cervical esophagus are the most common and can be further classified by zones [1,2]. From the clavicle to the cricoid cartilage is zone I, and from the level of the cricoid to the mandibular angle is zone II, where more than 80% of cervical neck injuries occur [2]. Zone III extends to the base of the skull [2]. The cervical esophagus is contained between the trachea and spine anteriorly and posteriorly, with the carotid sheath on either side, making this region of high importance [1]. Any complete perforation would result primarily in retro-esophageal extravasation due to the lateral attachment to the prevertebral fascia [3]. Here, the carotid arteries are the biggest concern, as otherwise blood is supplied by branches of the thyroid arteries [3]. Injuries to the thoracic esophagus are less common, but confer the highest mortality due to the approximation to vital structures and vessels, as well as a high risk of mediastinal sepsis [1,3]. The difference in transmural pressure within the thoracic cavity worsens existing perforations [3]. Finally, injuries to the abdominal esophagus are the least common, but they carry a high risk of complications and poor wound healing due to poor vascularization and a lack of the serosal layer [1].

Approximately 15% of injury is the result of spontaneous rupture in the setting of Achalasia, Boerhaave syndrome or strictures, or foreign body or caustic substance ingestion [1]. Some iatrogenic EIs are traumatic and result from blunt wounds or penetration, but the majority of iatrogenic EIs result from intraoperative or endoscopy procedures [1]. Previous studies have observed that gunshot wounds are the most common cause of penetrating traumatic EIs, accounting for 47-75% of traumatic EIs [1-3]. Stab wounds follow close behind, accounting for 15% of penetrating EIs in the United States; however, the prevalence of stab wounds versus gunshot wounds varies depending on region and country [1-3]. Even in high-influx trauma centers, EIs account for 1-6% of all trauma cases per year, and, overall, the incidence of traumatic EIs is very low worldwide [1,4]. Around 90% of the penetrating EIs involve the shallow cervical region [2]. Deep cervical EIs and penetrating thoracic EIs are very rare [2]. However, previous studies have demonstrated a higher risk of thoracic EIs by three times in males and five times in African Americans [3].

Herein, we present a case of a patient who suffered multiple stab wounds to the left side of his neck laterally and was incidentally found to have a thoracic esophageal perforation and superficial mucosal tears, which were repaired successfully endoscopically.

## Case presentation

A 30-year-old male with no past medical history was brought to the Emergency Department by ambulance after suffering multiple stab wounds to the left side of his neck and forearm. On arrival, he was hypotensive (107/70 mmHg), tachycardic (150 beats per minute), and saturating 100% on room air. His Glasgow coma scale was 3, for which he was intubated, and he was also noted to have active arterial bleeding with an expanding hematoma over the left neck. A focused echo and limited focus abdominal bedside ultrasound (FAST) examination did not identify free fluid in the hepatorenal, splenorenal, suprapubic, or pericardial spaces.

**Figure 1:**
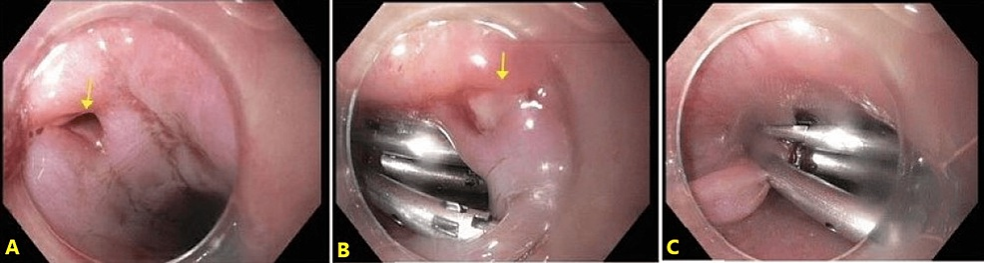
An esophagogastroduodenoscopy demonstrating esophageal perforation and mucosal tear repaired with endo-clips. (A) The arrow indicates an esophageal perforation in the upper esophagus, approximately 20 cm from the incisors. (B) An arrow indicates a mucosal tear in the upper third of the esophagus adjacent to the esophageal perforation after endo-clip placement. (C) The esophageal perforation in the upper esophagus, which was successfully closed with endo-clips.

He was subsequently brought to the operating room to control the bleeding, where he underwent a left neck exploration and repair of the left common carotid with a polytetrafluoroethylene (PTFE) graft and ligation of the left internal jugular vein. He was diagnosed with transection of the left common carotid artery, transection of the left internal jugular vein, and transection of the left vagus nerve; however, an incidental EI was noted. Intra-operatively, the patient also underwent a bronchoscopy and an esophagogastroduodenoscopy (EGD), which showed air bubbles at 22 cm; After 24 hours of the surgery, he was taken for an additional EGD to further assess and treat the esophageal defect. A perforation 20 cm from the incisors in the proximal esophagus was noted (Figure 1).

This was repaired with hemostatic clips. A non-bleeding mucosal tear was also found in the upper third of the esophagus adjacent to the esophageal perforation, also repaired endoscopically with hemoclips. Both defects were very small and less than 1 cm. The patient was extubated the next morning, and later that day, he underwent a barium esophagram, which showed no evidence of residual esophageal extravasation, indicative of a successful endoscopic repair. Approximately 72 hours later, significant swelling and erythema were noted at the medial aspect of the left side of the neck, and the patient was febrile with a maximum temperature of 40.3 degrees Celsius. A computed tomography (CT) revealed a subcutaneous collection concerning for hematoma versus abscess with inflammatory changes around the left supraclavicular fossa and at the base of the neck on the left. There was also subcutaneous emphysema within the neck, extending inferiorly into the chest wall, and a mass effect from the soft tissue collection causing tracheal deviation. The patient was maintained on intravenous metronidazole, vancomycin, and piperacillin-tazobactam since admission. At the discretion of the surgical team, he was taken back to the operating room for exploration of the neck and to check the integrity of the graft. A washout and evacuation of a hematoma located at the base of the neck, as well as an evolving abscess found in the supraclavicular fossa, was performed. Intra-operatively, a repeat EGD and esophagram were performed to evaluate the anatomic reconstruction and assess for further mucosal injury. Five hemostatic clips were noted in the upper esophagus without evidence of defect or laceration after 50 mL of contrast media injection. The surgical team dissected the left side of the neck and filled the field with sterile water, and subsequently air was insufflated through the endoscope into the esophagus to evaluate for communication. No air bubbles were noted during the examination.

The patient tolerated the procedures well. No further blood transfusions were warranted based on his clinical improvement. He was slowly advanced to a regular diet, remained hemodynamically stable, and was discharged with outpatient follow-up. He was ultimately referred to a Swallow Center for ongoing therapy. However, one month following discharge, he was seen in the Surgical Clinic with no dysphagia, odynophagia, hoarseness, shortness of breath, or cough. He continued to tolerate a regular diet.

## Discussion

EIs are difficult to diagnose because the presenting symptoms mimic other conditions and in the setting of trauma can be masked by other equally as sinister injuries to surrounding structures, such as the internal jugular vein, carotid vein, vocal cords, or thoracic blood vessels [1,2]. Specifically, upper and middle thoracic EI symptoms are non-specific and usually systemic, such as hypotension or bradycardia, and thus diagnosis is commonly missed or delayed, as those symptoms can easily be attributed to the penetration of other adjacent structures [1,2,5]. Chest pain is predominant in more than 70% of patients, regardless of the region involved [1,3]. The Mackler triad is when chest pain is accompanied by vomiting and subcutaneous emphysema and is seen in only one out of every seven cases [1,3]. A systolic “crunching” noise over the precordium is known as the Hamman sign and is seen in up to 50% of cases [1].

Because there are only a few comprehensive reports on EIs, protocols to guide diagnosis and treatment have not been fully established [2]. If time permits, chest radiographs can reveal acute perforation, although subcutaneous emphysema and mediastinal widening may not appear on imaging until hours later [1]. Often, radiographic evidence may take up to 12 hours to appear following injury, and therefore prompt diagnosis should be made based on history, clinical suspicion, and serial examinations [1]. Esophageal imaging allows for rapid detection of anatomical defects and extravasation [1]. Additionally, various types of contrast media, such as high osmolality agents, water-soluble agents, and barium sulfate can be useful in the detection of early perforation [1]. However, 10% of esophagograms show false-negative results if there is significant esophageal inflammation and/or edema present [1,4]. For this reason, CT is more frequently utilized as a first- or second-line imaging modality and can be especially useful where there is high clinical suspicion for EI despite a negative esophagogram [1,4]. CT with contrast allows for identification of esophageal wall thickening, edema, gas and fluid, and abscess formation or necrotizing lesions, as well as compromised vascular and respiratory structures [1,4]. Although less commonly utilized, flexible esophagoscopy is the most useful for direct visualization after traumatic EIs [3]. A cross-sectional analysis of Trauma Centers in Pennsylvania demonstrated that diagnostic endoscopy was used in only 10.4% of traumatic EIs [3].

The American Association for the Surgery of Trauma (AAST) developed a severity classification for EIs [1]. A grade I EI is a contusion, hematoma, or partial thickness tear [1], grade II is a laceration involving less than 50% circumference, and grade III is a laceration involving more than 50% circumference [1]. If less than 2 cm of the tissue or vasculature is damaged, it is considered grade IV, and if more than this, it is classified as grade V [1]. The treatment varies based on the severity and location of the EI; however, to our knowledge, only one other case report describes a repair exclusively via endoscopy [6]. Our patient was diagnosed with an EI via endoscopy, which was also repaired exclusively via endoscopy using hemoclips, making this case a rarity. The traditional approach to EIs is through surgical repair, although for many cervical penetrations, conservative management is accepted [1,2]. If a patient has a contained leak with mild symptoms and/or minimal evidence of sepsis, conservative management is warranted regardless of the region affected; however, the longer the delay until diagnosis, the higher the chance of infection and necrosis [1,2]. For those undergoing surgical repair, previous studies have not demonstrated any significant differences in outcomes or complications in those undergoing resection, anastomosis, diversion, and/or drainage [5]. Finally, when defects persist despite primary repair or when primary repair is not feasible, a diverting cervical esophagostomy should be performed [1].

Early diagnosis of traumatic EIs can minimize the potential of severe complications, such as mediastinitis, pyothorax, necrosis, abscess formation, and fistulation [1,2]. Overall, mortality can range from 20% to 50% in EIs; however, if a perforation goes undetected, the mortality rate can exceed 90% [3]. Patients with no injury to surrounding structures, particularly vessels, and those with cervical injuries carry better prognosis [1,3]. Trauma to the thoracic esophagus carries a higher mortality, as gastrointestinal contents can extravasate into the pleural cavity as well as the mediastinum and is surrounded by larger blood vessels [3]. Despite having a thoracic EI, our patient fortunately did well with the endoscopic repair and was able to tolerate a diet prior to discharge. No further blood transfusions were warranted based on his clinical improvement. He was ultimately referred to a Swallow Center for ongoing therapy, but when he returned to the clinic, he was asymptomatic.

## Conclusions

EI can carry a high mortality rate, especially when the thoracic region is affected. Early detection is imperative to minimize mortality, morbidity, and associated complications. Uncommonly, endoscopy is used to diagnose an EI and rarely used to treat one. Our patient was diagnosed and treated endoscopically for a thoracic EI successfully. This approach was less invasive than surgical treatment and was not correlated to any post-procedural complications, even in a patient who presented with hemorrhagic shock. In these instances, if exploratory surgery is not warranted, one can consider endoscopic intervention as an initial approach to patients who may be more clinically stable.
